# Sexual and Reproductive Health and Education of Adolescents during COVID-19 Pandemic, Results from “Come Te La Passi?”—Survey in Bologna, Italy

**DOI:** 10.3390/ijerph19095147

**Published:** 2022-04-23

**Authors:** Marco Montalti, Aurelia Salussolia, Alice Masini, Elisa Manieri, Flavia Rallo, Sofia Marini, Marta Agosta, Martina Paternò, Michela Stillo, Davide Resi, Federica Guaraldi, Davide Gori, Laura Dallolio

**Affiliations:** 1 Department of Biomedical and Neuromotor Sciences, University of Bologna, 40138 Bologna, Italy; marco.montalti7@studio.unibo.it (M.M.); aurelia.salussolia@studio.unibo.it (A.S.); flavia.rallo@studio.unibo.it (F.R.); davide.gori4@unibo.it (D.G.); laura.dallolio@unibo.it (L.D.); 2 Department of Pediatrics, Pediatric Medical School, University of Bologna, Via Massarenti 11, 40138 Bologna, Italy; elisa.manieri3@studio.unibo.it; 3 Department for Life Quality Studies, University of Bologna, 47921 Rimini, Italy; sofia.marini2@unibo.it; 4 Department of Public Health, Bologna Local Health Authority, 40124 Bologna, Italy; agosta.marta@ausl.bologna.it (M.A.); martina.paterno@ausl.bologna.it (M.P.); michela.stillo@ausl.bologna.it (M.S.); davide.resi@ausl.bologna.it (D.R.); 5 IRCCS Istituto delle Scienze Neurologiche di Bologna, 40139 Bologna, Italy; federica.guaraldi@yahoo.it

**Keywords:** sexual health, reproductive health, SRH, sexual education, information sources, sexual debut, SARS-CoV-2, adolescents, health care providers

## Abstract

Social distancing measures adopted to face the COVID-19 pandemic had a detrimental impact on adolescent education and their interaction with peers and adults, secondary to the limitation of school and recreational activities, with repercussions on social and sexual life. The “Come te la passi?” (“How is it going?”) study, performed in the Metropolitan City of Bologna (Italy), aimed at investigating the type of information sources adopted by adolescents for their sexual and reproductive health (SRH) knowledge and education, the age of their sexual debut, and the way in which the COVID-19 pandemic affected their relationships and sexual life in order to help local health care professionals and educators designing SRH education programs. A purposely designed online survey was administered during the COVID-19 pandemic to 378 high school students (age > 14 yo) in July 2021. Based on the study results, the most common source of SRH education was the web, followed by peers (friends). A total of 61.3% of 17-year-olds already had sexual intercourse, and 90% of 15-year-olds had experienced romantic or sexual attraction. For 58.2% of the adolescents, the COVID-19 pandemic had negative effects on their relationships/sexual life. The current research emphasizes the need to involve health care professionals and educators in structured programs to promote SRH education tailored to adolescents’ needs and started from early ages.

## 1. Introduction

In February 2020, the world was disrupted by the SARS-CoV-2 infection, responsible for the COVID-19 disease, first defined by the World Health Organization (WHO) as a Public Health Emergency of International Concern [1] and then, on 11 March 2020, as a global pandemic [2].

From the end of February 2020, Italy, like the majority of countries worldwide, put in place several measures to limit the spread of the COVID-19 disease, including social distancing and the use of facial masks as a means of individual protection. Then, on 11 March, a national lockdown was declared [3]. This implied the restriction of individual movements and the closure of public places, business facilities, and schools. Starting from Autumn 2020, children’s education was impacted by distance learning [4]. These measures were responsible for the discontinuity of the educational service and the dramatic reduction of young people’s social and physical relationships.

According to the United Nations International Children’s Emergency Fund (UNICEF), the Organization for Economic Cooperation and Development (OECD), and the European Centre for Disease Prevention and Control (ECDC), young people have been significantly impacted in physical, psychological, and social terms by the COVID-19 pandemic [5,6,7]. Sexual and reproductive health (SRH) was also deeply affected, not only because adolescence is a crucial age for sexual development through personal sexual experiences [8] but also in terms of access to and use of SRH care, preventive and screening services, abortion care, and SRH education programs [9].

SRH is defined by the WHO as a state of physical, emotional, mental, and social well-being in relation to sexuality. It is therefore not merely the absence of disease, dysfunction, or infirmity. SRH requires a positive and respectful approach to sexuality and sexual relationships. For SRH to be achieved and maintained, the sexual rights of all people must be respected, protected, and fulfilled [10]. The importance of achieving these goals has been underlined with the inclusion of specific targets for SRH within the 2030 Sustainable Development Goals Agenda [11].

SRH education is therefore an essential element in achieving these goals. In particular, according to the United Nations Population Fund, SRH education programs are all more effective when they are based on the Comprehensive Sexuality Education (CSE) approach, a rights-based and gender-transformative approach [12]. The CSE approach consists of programs taught over several years, integrating age-appropriate information (human development, anatomy, and reproductive health, as well as information on contraception, childbirth, and sexually transmitted infections) that takes into account young people’s developmental capacities [13]. In order to create an enabling environment for SRH, it is necessary to include different levels: individual level (e.g., empowering girls); relationship level (e.g., building parental support); and community level (e.g., transforming gender and other social norms) [14].

Most adolescents use formal (i.e., health care providers (HCP) and/or educators) and informal sources of education (i.e., web, peers, and/or family members to interpret an increasing range of sexual experiences). [15]. Several studies carried out before the pandemic showed that SRH education at school is effective in improving the knowledge and health of young people [16] but also highlighted gaps and critical issues in Italy, where SRH and education programs are not standardized at a national level and were judged below European standards, thus requiring implementation [17,18,19].

Given these premises, understanding the information sources on SRH knowledge, observing the timing of first relational and sexual experiences, and understanding how the COVID-19 pandemic has affected the perception of romantic/sexual relationships in adolescents at a local level appear to be of primary importance for their potential consequences in a post-pandemic era. The aim of the present study was to analyze the key elements of SRH and education among adolescents in the Metropolitan City of Bologna (Italy) in order to help HCPs and educators in designing SRH education programs, adapting them to specific local targets and needs.

## 2. Materials and Methods

### 2.1. Study Design and Setting

We performed a cross sectional study among adolescents attending high schools in the Metropolitan City of Bologna. The study was approved by the University of Bologna Ethics Committee on 14 July 2021 (Prot. N. 170328) and by the boards of the schools in which the questionnaires were administered. The study was conducted following the Declaration of Helsinki. The data were collected using a purposely designed self-administered questionnaire delivered in July 2021 to parents/guardians of adolescents (14 years or older) attending high schools in the Metropolitan City of Bologna. The Bologna Local Health Authority School-Unit, which was responsible for managing SARS-CoV-2 outbreaks within schools, disseminated the online questionnaire to school contact persons/heads, who then emailed the questionnaire to parents/guardians. All of the schools of the Metropolitan City of Bologna, both public and private, were invited.

The email sent to parents/guardians included a short text introducing the study, written by the health authority staff, and the link to the online questionnaire. The first section required parents/guardians to fill in. Before the beginning of the second section, a text appeared asking parents/guardians to respond together with their children to the next section (if under 18) or asking them to let their children respond to the following section independently (if over 18).

The questionnaire collected socio-demographic data of interest (i.e., gender, age, school attended) and explored some specific domains of interest identified in the National Survey of Adolescents and Young Adults on Sexual Health Knowledge, Attitudes and Experiences (2003) conducted in the USA [20]. Nine questions referring to sources of sex education were based on a four-level Likert scale (from 0 = “no information received” to 4 = “lots of information received”), while seven closed yes–no (dichotomic) questions were aimed at investigating adolescents’ sexual experiences. Furthermore, using a five-level Likert scale (1 = “very negatively”; 5 = “very positively”), we asked study participants how much the COVID-19 pandemic has affected their romantic/sexual relationships. An English version of the survey instrument can be found in the Supplementary Materials (see Supplementary Materials, Questionnaire instrument).

### 2.2. Data Analysis

The data were anonymized by assigning a unique identification number to each participant and electronically stored on a secured file safeguarded by a password. Non-parametric variables were described as absolute and relative frequencies; parametric variables were described as means with standard deviations.

Determinants of the worsening of romantic/sexual relationships following the COVID-19 pandemic were assessed by multivariate analysis. In addition, a backward stepwise logistic-regression analysis was run to define the variables to be included in the final multiple logistic regression model, according to the principles of parsimony and biological plausibility. The results of the multivariate analyses were presented as odds ratio (OR) with standard error (SE) and 95% Confidence Intervals (95% CI). The data were collected using Microsoft Excel (Microsoft Corporation). All analyses were carried out using Stata Statistical Software 15 (StataCorp, College Station, TX, USA).

## 3. Results

A total of 378 adolescents over 14 yo (males 60.1%) attending 24 high schools in the Metropolitan City of Bologna were enrolled in the study. The main sample demographic features are reported in Table 1.

### 3.1. Sources of Sex Education

**Figure 1:**
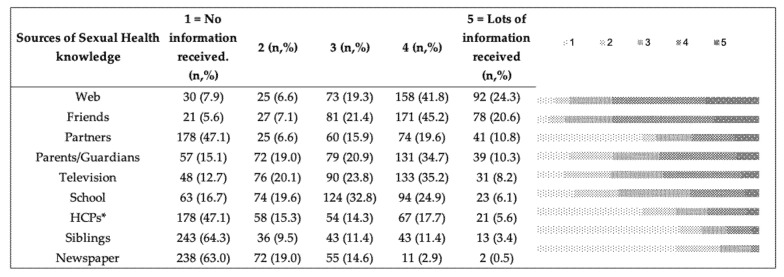
Sources of information chosen by high school students of the Metropolitan City of Bologna for SRH knowledge. * HCPs = Health Care Professionals.

Overall, 66.1% (*n* = 250) of the adolescents reported to have found useful information (score 4 and 5 on the Likert scale) on the web, and 65.8% (*n* = 249) from friends (Figure 1). Schools and HCPs were considered useful sources of information by 31.0% (*n* = 117) and 23.3% (*n* = 88) of them, respectively. Only 3.4% (*n* = 13) of the participants referred to newspapers as sources of SHR information.

### 3.2. Sexual Experiences

Overall, 56 (73.7%) of 14-year-old adolescents answered that they had already felt romantic and sexual attraction towards another person, a rate that rose to 91.3% (*n* = 21) when considering the over-19s (Table 2). In particular, 39.5% (*n* = 30) of the 14-year-olds, 50% (*n* = 35) of the 15-year-olds, and 78.3% (*n* = 18) of the over-19s reported to have already kissed another person. Moreover, 7.9% (*n* = 6) of the 14-year-olds and 56.5% (*n* = 13) of the over-19s reported to have been involved in a relationship; for 25.0% (*n* = 19) of the 14-year-old adolescents and 34.8% (*n* = 8) of the 19-year-olds, it occurred before the pandemic. Finally, 13.2% (*n* = 10) of the 14-year-olds, 61.3% (*n* = 46) of the 17-year-olds, and 56.5% (*n* = 13) of the over-19s had already experienced intimate or sexual experiences.

When interviewed about the impact of the COVID-19 pandemic on relational life, 58.2% (*n* = 220) of the adolescents answered ‘negatively’ or ‘very negatively’, while 13.3% (*n* = 50) answered ‘positively’ or ‘very positively’.

### 3.3. Predictors of Worsening of the Romantic/Sexual Relationships

We compared respondents who reported a worsening in their romantic/sexual relationship due to the COVID-19 pandemic and the aforementioned restrictive measures to demographic characteristics and romantic/sexual experiences they had. The results of the multivariate analysis and adjusted multivariate analysis are reported in Supplementary Material Table S1a,b.

According to our results, having had romantic/sexual experiences was associated with a significant worsening of romantic/sexual relationships (*p* < 0.05).

## 4. Discussion

We have reported the results of a survey on the SRH education and changes in romantic/sexual relationships during the COVID-19 pandemic in adolescents of the Metropolitan City of Bologna.

Based on our data, most adolescents refer to the web as a source of information on SRH, followed by friends and parents/guardians. It is not surprising that adolescents increasingly turn to digital media to answer their health questions, including those related to SRH, as the great majority of them are connected online via social media and mobile applications [21]. The online health information seeking behavior of young people, especially adolescents, has been the subject of many studies over the last decade [22,23,24,25], which report an increasing number of them going online to find health information and many of them considering the web as a primary source for such information [26]. From this perspective, it becomes strategic for public health interventions and informational campaigns, designed by HCPs and educators, to be tailored to reflect the ways in which children and adolescents navigate digital health—and, specifically, SRH—information [21].

In our sample, the two least used sources by the participants were HCPs together with partners. In a recent review on the effectiveness of SRH education programs, Corcoran et al. found that adolescents seek external sources of education, e.g., peers and media, when the content provided by educators or HCPs is deemed irrelevant or the education is perceived as partial [27]. The fact that partners are not seen as useful sources for SRH education may indicate that adolescents are not facing this issue with the people they interact with sexually and romantically. Recognizing and understanding adolescents’ needs, perceptions, and experiences is of utmost importance to improve SRH education programs and promote healthy sexual development in adolescents. Indeed, adolescents seem to judge the quality of SRH education on the basis of the open-mindedness and knowledge of the educators or HCPs, the relevance of the content, and their comfort with the educational environment [28,29,30,31]. One way to address these needs is through the adoption and planning of CSE programs that follow evidence-based guidelines and are led by HCPs and educators with ongoing training [32].

Moreover, many adolescents had their sexual debut at a very early age, in line with other Italian and international studies [33,34,35]. This finding underlines the potential importance of starting SRH education programs from the first year of high school in order to address as many adolescents as possible before or during their first romantic/sexual experience and to continue the programs throughout time with a CSE approach. This was also underlined by another recent study conducted among Italian adolescents attending the first year of high school, which showed that the general level of knowledge about SRH was inadequate among half of the adolescents [36].

Finally, the worsening of romantic/sexual relationships due to the COVID-19 pandemic was reported by more than half of the participants. A similar result was found in an Australian study assessing the psychological impact and the types of relationships of adolescents with their peers during the pandemic. Most of the respondents reported having felt less connected to their friends and a worsening of their romantic relationships, despite the use of digital systems to stay connected [37].

Our study presents some limitations that should be disclosed. Firstly, school participation in our study was suboptimal, potentially affecting representativeness, as previously experienced in other studies [36,38]. Furthermore, the cross-sectional study design provides a static picture of the period in which the data were collected. The non-randomly selected participants were enrolled via email; thus, (a) it is not possible to calculate a survey participation rate, and (b) it is not clear which subset of the adolescent population the survey participants represent. Moreover, since the second section of the questionnaire (in cases of children < 18 years old) could be filled together with their parents/guardians, there might be some social desirability bias in the answers due to the presence of the latter. Finally, in the second section relating to the source of information, students with no partners or without siblings could not answer ‘non-applicable’, leading to a possible underestimation of the data. Concurrently, the main strengths of the study are the large sample of enrolled subjects and schools, the absence of operator influence on questionnaire-filling, and the homogeneous distribution of adolescents for age. Future studies should also investigate the quality and utility of the information received regarding sexual health that this questionnaire did not investigate.

Despite these limitations, our findings contribute to an understanding of the most used information sources about SRH by adolescents in the Metropolitan City of Bologna, at what age they had their sexual debut, and how their relationships worsened in the context of the current COVID-19 pandemic.

## 5. Conclusions

The “Come te la Passi” study has observed that adolescents from the Metropolitan City of Bologna seek information and receive education on SRH mainly by accessing the web and discussing with friends, while partners and HCPs were the least relied upon sources of information. More than half reported sexual debut before the age of 17, and most of them experienced a worsening of their romantic/sexual life during and caused by the COVID-19 pandemic. This survey could be of help for HCPs and educators in defining targeted CSE programs based on local adolescents’ needs.

Future research studies focused on CSE programs should be based on a co-designed methodology with a bottom-up approach in order to increase the effectiveness and satisfaction of the participants.

## Figures and Tables

**Table 1:** Main sample demographic and anthropometric features (*n* = 378).

Feature		N (%) or Mean ± SD
Gender	Male	227 (60.1)
	Female	148 (39.2)
	Other	3 (0.8)
Age (years)	14	76 (20.1)
	15	70 (18.5)
	16	94 (24.9)
	17	75 (19.8)
	18	40 (10.6)
	≥19	23 (6.1)

**Table 2:** Sexual experiences among adolescents in the Metropolitan City of Bologna (a) and changes in the romantic/sexual relationships during the COVID-19 pandemic (b).

**a.**																	
		**Romantic/Sexual Experience (*n*, %)**															
		**Have You Ever Felt a Romantic/Sexual Attraction for Another Person?**		**Have You Ever Kissed Someone Romantically?**		**Have You Ever Been with Someone in an Intimate or Sexual Way?**		**Have You Ever Been in a Romantic/Sexual Relationship?**		**Number of Partners You Have Been with in a Romantic/Sexual Relationship**						**Were You in a Romantic/Sexual Relationship before the COVID-19 Pandemic?**	
		**Yes**	**No**	**Yes**	**No**	**Yes**	**No**	**Yes**	**No**	**1**	**2**	**3**	**4**	**5**	**≥6**	**Yes**	**No**
Age (years)	14 (*n* = 76)	56 (73.7)	20 (26.3)	30 (39.5)	46 (60.5)	10 (13.2)	66 (86.8)	6 (7.9)	70 (92.1)	8	1	1	0	0	0	19 (25.0)	57 (75.0)
	15 (*n* = 70)	63 (90.0)	7 (10.0)	35 (50.0)	35 (50.0)	13 (18.6)	57 (81.4)	9 (12.9)	61 (87.1)	12	3	1	1	0	0	7 (10.0)	63 (90.0)
	16 (*n*= 94)	77 (81.9)	17 (18.1)	56 (59.6)	38 (40.4)	34 (36.2)	60 (63.8)	27 (28.7)	67 (71.3)	23	3	2	1	1	4	20 (21.3)	74 (78.7)
	17 (*n* = 75)	66 (88.0)	9 (12.0)	53 (70.7)	22 (29.3)	46 (61.3)	29 (38.7)	42 (56.0)	33 (44.0)	26	7	5	4	2	1	22 (29.3)	53 (70.7)
	18 (*n*= 40)	32 (80.0)	8 (20.0)	30 (75.0)	10 (25.0)	22 (55.0)	18 (45.0)	21 (52.5)	19 (47.5)	13	1	5	6	1	2	13 (32.5)	27 (67.5)
	≥19 (*n* = 23)	21 (91.3)	2 (8.7)	18 (78.3)	5 (21.7)	13 (56.5)	10 (43.5)	13 (56.5)	10 (43.5)	5	4	2	0	0	2	8 (34.8)	15 (65.2)
Total	378	315 (83.3)	63 (16.7)	222 (58.7)	156 (41.3)	138 (36.5)	240 (63.5)	118 (31.2)	260 (68.8)							89 (23.5)	289 (76.5)
				**b.**													
								***n* (%)**									
				**Impact of the COVID-19 pandemic on romantic/sexual relationship**		1 (very negative)		113 (29.9)									
						2 (negative)		107 (28.3)									
						3 (neither negative nor positive)		108 (28.6)									
						4 (positive)		35 (9.3)									
						5 (very positive)		15 (4.0)									

## Data Availability

The data presented in this study are available on request from the corresponding author. The data are not publicly available due to ethical and privacy reasons.

## Supplementary Materials

The following supporting information can be downloaded at: https://www.mdpi.com/article/10.3390/ijerph19095147/s1, Questionnaire instrument. Table S1. Predictors of a worsening in the romantic/sexual relationships following the COVID-19 pandemic according to multivariate analysis (a), adjusted multivariate analysis for a backward stepwise analysis and principle of parsimony and biological plausibility (b).

## References

[1] World Health Organization (2020). Timeline: WHO’s COVID-19 Response.

[2] (2020). WHO Director-General’s Opening Remarks at the Media Briefing on COVID-19. https://www.who.int/director-general/speeches/detail/who-director-general-s-opening-remarks-at-the-media-briefing-on-covid-19---11-march-2020.

[3] Decree of the President of the Council of Ministers 11 March 2020. Further Implementing Provisions of Decree-Law No. 6 of 23 February 2020 on Urgent Measures for the Containment and Management of the Epidemiological Emergency from COVID-19, Applicable Throughout the Country. http://www.trovanorme.salute.gov.it/norme/dettaglioAtto?id=73643.

[4] Decree of the President of the Council of Ministers 3 November 2020. https://www.gazzettaufficiale.it/eli/id/2020/11/04/20A06109/sg.

[5] UNICEF The Impact of COVID-19 on the Mental Health of Adolescents and Youth. https://www.unicef.org/lac/en/impact-covid-19-mental-health-adolescents-and-youth.

[6] OECD Supporting Young People’s Mental Health through the COVID-19 Crisis. https://read.oecd-ilibrary.org/view/?ref=1094_1094452-vvnq8dqm9u&title=Supporting-young-people-s-mental-health-through-the-COVID-19-crisis.

[7] ECDC COVID-19 in Children and the Role of School Settings in Transmission—Second Update. https://www.ecdc.europa.eu/sites/default/files/documents/COVID-19-in-children-and-the-role-of-school-settings-in-transmission-second-update.pdf.

[8] Marino C., Vieno A., Lenzi M., Santinello M. (2014). Time trends in adolescent sexual behaviour in Italy. Sex Health.

[9] Lindberg L.D., Bell D.L., Kantor L.M. (2020). The Sexual and Reproductive Health of Adolescents and Young Adults during the COVID-19 Pandemic. Perspect. Sex. Reprod. Health.

[10] Sexual and Reproductive Health and Research (SRH). https://www.who.int/teams/sexual-and-reproductive-health-and-research/key-areas-of-work/sexual-health/defining-sexual-health.

[11] Transforming Our World: The 2030 Agenda for Sustainable Development. https://sdgs.un.org/2030agenda.

[12] Bakaroudis M., Blum R., Hopkins J. The Evaluation of Comprehensive Sexuality Education Programmes: A Focus on the Gender and Empowerment Outcomes. https://www.unfpa.org/sites/default/files/pub-pdf/UNFPAEvaluationWEB4.pdf.

[13] Comprehensive Sexuality Education. https://www.unfpa.org/comprehensive-sexuality-education#readmore-expand.

[14] Svanemyr J., Amin A., Robles O.J., Greene M.E. (2015). Creating an enabling environment for adolescent sexual and reproductive health: A framework and promising approaches. J. Adolesc. Health.

[15] Fortenberry J.D. (2014). Sexual Learning, Sexual Experience, and Healthy Adolescent Sex. New Dir. Child Adolesc. Dev..

[16] Capuano S., Simeone S., Scaravilli G., Raimondo D., Balbi C. (2009). Sexual behaviour among Italian adolescents: Knowledge and use of contraceptives. Eur. J. Contracept. Reprod. Health Care.

[17] Poscia A., La Milia D.I., Lohmeyer F., Teleman A.A., de Waure C., Ricciardi W. (2015). Sexual behaviours and preconception health in Italian university students. Ann. Dell’istituto Super. Sanita.

[18] Bogani G., Cromi A., Serati M., Monti Z., Apolloni C., Nardelli F., di Naro E., Ghezzi F. (2015). Impact of School-Based Educational Programs on Sexual Behaviors Among Adolescents in Northern Italy. J. Sex Marital Ther..

[19] Drago F., Ciccarese G., Zangrillo F., Gasparini G., Cogorno L., Riva S., Javor S., Cozzani E., Broccolo F., Esposito S. (2016). A Survey of Current Knowledge on Sexually Transmitted Diseases and Sexual Be-haviour in Italian Adolescents. Int. J. Environ. Res. Public Health.

[20] (2003). National Survey of Adolescents and Young Adults: Sexual Health Knowledge, Attitudes and Experiences. https://www.kff.org/hivaids/report/national-survey-of-adolescents-and-young-adults/.

[21] Center on Media and Human Development (2015). Teens, Health, and Technology: A National Survey.

[22] Borzekowski D.L., Fobil J.N., Asante K.O. (2006). Online access by adolescents in Accra: Ghanaian teens’ use of the internet for health information. Dev. Psychol..

[23] Eysenbach G., Miriam J.M., Andrew J.F. (2008). Credibility of Health Information and Digital Media: New Perspectives and Implications for Youth. Digital Media, Youth, and Credibility.

[24] Harvey K.J., Brown B., Crawford P., Macfarlane A., McPherson A. (2007). ‘Am I normal?’ Teenagers, sexual health and the internet. Soc. Sci. Med..

[25] Ybarra M.L., Emenyonu N., Nansera D., Kiwanuka J., Bangsberg D.R. (2008). Health information seeking among Mbararan adolescents: Results from the Uganda Media and You survey. Health Educ. Res..

[26] Gray N.J., Klein J.D., Noyce P.R., Sesselberg T.S., Cantrill J.A. (2005). The Internet: A window on adolescent health literacy. J. Adolesc. Health.

[27] Corcoran J.L., Davies S.L., Knight C.C., Lanzi R.G., Li P., Ladores S.L. (2020). Adolescents’ perceptions of sexual health education programs: An integrative review. J. Adolesc..

[28] Barbagallo M., Boon H. (2012). Young people’s perceptions of sexuality and relationships education in Queensland schools. Aust. Int. J. Rural Educ..

[29] Brown G., Sorenson A., Hildebrand J. (2012). How they got it and how they wanted it: Marginalised young people’s perspective on their experiences of sexual health education. Sex Educ..

[30] Byers E.S., Dawn Hamilton L., Fisher B. (2017). Emerging adults’ experiences of middle and high school sexual health education in New Brunswick, Nova Scotia, and Ontario. Can. J. Hum. Sex.

[31] Helmer J., Senior K., Davison B., Vodic A. (2015). Improving sexual health for young people: Making sexuality education a priority. Sex Educ..

[32] International Technical Guidance on Sexuality Education an Evidence-Informed Approach. https://www.unfpa.org/sites/default/files/pub-pdf/ITGSE.pdf.

[33] Panatto D., Amicizia D., Lugarini J., Sasso T., Sormani M.P., Badolati G., Gasparini R. (2009). Sexual behaviour in Ligurian (Northern Italy) adolescents and young people: Suggestions for HPV vaccination policies. Vaccine.

[34] Durda-Masny M., Jarząbek-Bielecka G., Szwed A., Hanć T., Czapla Z., Kaczmarek M. (2018). Trends over time in age at sexual debut among Polish women and underlying socio-economic determinants. Anthropol. Anz..

[35] Hansen B.T., Kjaer S.K., Arnheim-Dahlström L., Liaw K.L., Juul K.E., Thomsen L.T., Frederiksen K., Elfström K.M., Munk C., Nygård M. (2020). Age at first intercourse, number of partners and sexually transmitted infection prevalence among Danish, Norwegian and Swedish women: Estimates and trends from nationally representative cross-sectional surveys of more than 100,000 women. Acta Obstet. Gynecol. Scand..

[36] Brunelli L., Bravo G., Romanese F., Righini M., Lesa L., De Odorico A., Bastiani E., Pascut S., Miceli S., Brusaferro S. (2022). Sexual and reproductive health-related knowledge, attitudes and support network of Italian adolescents. Public Health Pract..

[37] Li S.H., Beames J.R., Newby J.M., Maston K., Christensen H., Werner-Seidler A. (2021). The impact of COVID-19 on the lives and mental health of Australian adolescents. Eur. Child Adolesc. Psychiatry.

[38] Visalli G., Picerno I., Vita G., Spataro P., Bertuccio M.P. (2014). Knowledge of sexually transmitted infections among younger subjects of the city of Messina (Sicily). J. Prev. Med. Hyg..

