# Supplementary material for: Sexual and Reproductive Health and Education of Adolescents during COVID-19 Pandemic, Results from “Come Te La Passi?”—Survey in Bologna, Italy
Source: Int J Environ Res Public Health. 2022 Apr 23;19(9):5147. doi: 10.3390/ijerph19095147 (PMC9102478; doi:10.3390/ijerph19095147)
Supplement: Supplementary file 1 [file ijerph-19-05147-s001.zip › ijerph-1629890-supplementary.pdf]

International Journal of
Environmental Research and Public Health

MDPI

Supplementary Material

Questionnaire Instrument

Section S1

Do you consent to the processing of your data?

- Yes
- No

Gender (son/daughter)?

- M
- F
- Other

Name of your child's school _________________
Class (in numbers, e.g., "2") _________________
Section (in letters, e.g., "A" or "BL") _________________

Section S2

We have some questions about your experiences. If you do not feel comfortable, you are free not to answer and proceed with the form. Thinking back to when you got information about relationships and sexual health, how much did you learn from (from 1 = "no information received" to 5 = "lots of information received"):

	1	2	3	4	5
Parents					
Siblings					
Web					
Friends					
Television					
School					
Health Care Professionals					

Have you ever experienced a romantic/sexual attraction to another person?

- Yes
- No

Have you ever kissed someone romantically?

- Yes
- No

Have you ever been with someone intimately or sexually?

- Yes
- No

Have you ever had a sexual relationship?

- Yes
- No

With how many people have you had sexual relations?

- 1
- 2
- 3
- 4

- 5
- 6
- more

Were you in a romantic/sexual relationship before the start of the pandemic?

- Yes
- No

Are you currently in a romantic/sexual relationship?

- Yes
- No

Has your romantic/sexual situation changed during the COVID19 pandemic?

- Yes
- No

How has the COVID pandemic affected your romantic/sexual relationships? (1 = "very negatively"; 5 = "very positively")

1	2	3	4	5

How old are you?

- 14
- 15
- 16
- 17
- 18
- 19
- more

Table S1. Predictors of a worsening in the romantic/sexual relationships following the COVID-19 pandemic according to multivariate analysis (a), adjusted multivariate analysis for a backward stepwise analysis and principle of parsimony and biological plausibility (b).

a.				
**Worsening of Romantic/Sexual Relationship Due to the COVID-19 Pandemic**	**OR**	**SE**	***p*-Value**	**95% C.I.**
Age	0.96	0.13	0.766	0.74–1.24
Gender (Female)	1.27	0.43	0.467	0.66–2.48
Romantic/sexual attraction experienced previously	0.18	0.26	0.227	0.01–2.91
Previous experience(s) of romantic/sexual kisses	1.30	1.06	0.752	0.26–6.49
Intimate/sexual experience(s) with someone else	1.55	1.20	0.574	0.34–7.08
Romantic/sexual relationship(s) experienced	1.91	1.26	0.325	0.53–6.94
Number of partners	0.90	0.11	0.377	0.71–1.14
In a romantic/sexual relationship before the COVID-19 pandemic outbreak	0.61	0.23	0.185	0.30–1.27
**b.**				
**Worsening of Romantic/Sexual Relationship Due to the COVID-19 Pandemic**	**OR**	**SE**	***p*-Value**	**95% C.I.**
Age	1.02	0.07	0.758	0.89–1.18
Gender (Female)	1.01	0.21	0.956	0.67–1.53
Romantic/sexual relationship(s) experienced	1.63	0.41	**0.049**	1.01–2.66
